# Biological Effects of Dietary Restriction on Alzheimer's Disease: Experimental and Clinical Investigations

**DOI:** 10.1111/cns.70392

**Published:** 2025-04-17

**Authors:** Zijiao Liu, Jun Zhang, Fei Jiang, Cong Liu, Yaping Shao, Weidong Le

**Affiliations:** ^1^ Key Laboratory of Liaoning Province for Research on the Pathogenic Mechanisms of Neurological Diseases The First Affiliated Hospital, Dalian Medical University Dalian China; ^2^ Clinical Research Center for Psychiatry Dalian Seventh People's Hospital Dalian China; ^3^ Interdisciplinary Research Center on Biology and Chemistry, Shanghai Institute of Organic Chemistry, Chinese Academy of Sciences Shanghai China; ^4^ Center for Clinical and Translational Research Shanghai University of Medicine and Health Sciences Shanghai China

**Keywords:** Alzheimer's disease, calorie restriction, cognitive decline, dementia, dietary restriction, intermittent fasting, treatment

## Abstract

**Backgrounds:**

Dementia can impose a heavy economic burden on both society and families. Alzheimer's disease (AD), the most prevalent form of dementia, is a complex neurodegenerative disease characterized by the abnormal deposition of extracellular amyloid β‐protein (Aβ) and the aggregation of intracellular Tau protein to form neurofibrillary tangles (NFTs). Given the limited efficacy of pharmacological treatment, scientists have already paid more attention to non‐pharmacological strategies, including dietary restriction (DR). DR refers to a nutritional paradigm aimed at promoting overall health by modifying the balance between energy consumption and expenditure. Studies have demonstrated that DR effectively extends the healthy lifespan, delays the aging process, and achieves promising results in the prevention and treatment of AD in preclinical studies.

**Methods:**

In this review we collected related studies and viewpoints by searching on PubMed database using the keywords. Most of the citations were published between 2015 and 2025. A few older literatures were also included due to their relevance and significance in this field.

**Results:**

We first provide a concise overview of the current therapeutic and preventive strategies for AD. Then, we introduce several specific DR protocols and their favorable effects on AD. Furthermore, the potential mechanisms underlying the benefits of DR on AD are discussed. Finally, we briefly highlight the role of DR in maintaining brain health.

**Conclusion:**

This review may offer valuable insights into the development of innovative non‐pharmacological strategies for AD treatment.

## Graphical abstract

This review explores the role of dietary restriction as a non‐pharmacological strategy in the prevention and treatment of Alzheimer's disease, focusing on its potential mechanisms and its association with AD pathologies.

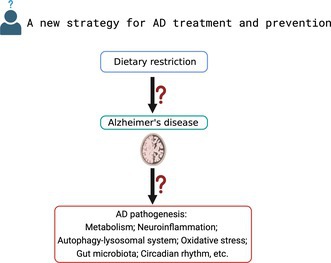

## 1 Introduction

Dementia is a general term for a group of disorders characterized by a progressive decline in cognitive functions, primarily affecting patients' memory and daily living abilities. According to the World Health Organization (WHO), over 55 million individuals worldwide are suffering from dementia, with 60% residing in low‐ and middle‐income countries [1]. Alzheimer's disease (AD), the most prevalent form of dementia, accounts for approximately 60%–80% of all cases and predominantly affects individuals over 65 years old [2]. AD is a multifaceted neurodegenerative disease, characterized by the abnormal deposition of extracellular amyloid β‐protein (Aβ) into amyloid plaques and the aggregation of intracellular Tau protein to form neurofibrillary tangles (NFTs) [3]. As research progresses, misfolding of proteins, dysfunction of autolysosome and mitochondria, oxidative stress, and neuroinflammation have emerged as pivotal mechanisms underlying the onset of AD [4, 5, 6, 7]. However, due to the intricate pathogenesis of AD, single‐target drugs often fail to achieve anticipated therapeutic outcomes. Consequently, researchers have gradually focused on non‐pharmacological interventions, particularly dietary restriction (DR) including calorie restriction (CR), intermittent fasting (IF), and ketogenic diet (KD), etc. [8, 9, 10] Current studies report that the adoption of a rational dietary regimen, characterized by precise timing, appropriate frequency, and balanced calorie intake, can significantly enhance overall health status, without incurring severe adverse effects [11].

It is generally believed that aging is a natural developmental trajectory that cannot be intervened. In 1928, Pearl proposed the “rate of living” aging theory, which posits that the lifespan of an organism is intrinsically linked to its metabolic rate, with the concomitant production of excessive free radicals that exert deleterious effect on cellular and tissue integrity [12]. DR can combat this process by modulating cellular metabolic rate and mitigating oxidative stress‐induced damage, thus playing an important role in the prevention and amelioration of aging‐related diseases, including AD [13, 14]. Over the past few decades, studies have shown that DR can effectively extend the healthy lifespan across various species, including flies, rodents, and non‐human primates, delay the aging process, and have achieved progress in the laboratory settings for the prevention and treatment of AD [10]. Nevertheless, the intricate mechanism underlying the favorable effects of DR and the extent to which it can alleviate AD‐related symptoms remain pressing issues that warrant further elucidation. This review primarily focuses on DR and its potential mechanisms by which DR may exert its beneficial effects on AD pathology, thereby providing new insights and perspectives for the prevention and therapeutic management of AD.

## 2 General Review of Treatment and Prevention of Alzheimer's Disease

AD is the most prevalent neurodegenerative disease (ND), which can be divided into three stages: preclinical AD, mild cognitive impairment (MCI), and dementia [2]. As life expectancy extends with the progress of society, the number of patients suffering from AD is rising globally and is expected to increase to 139 million by 2050, given that age is the most significant risk factor for both AD and dementia [15, 16]. Besides, gender also plays a role in the onset of AD, with statistics indicating that women have higher incidence rates of dementia compared to men [17]. However, scientists have not yet reached a consensus on how to explain this difference, which may be attributed to factors such as education attainment or hormonal exposure [18]. In addition to these uncertain risk factors, the pathogenesis of AD is complex, leading to unsatisfactory treatment outcomes. In this section, we will discuss current treatment approaches and potential prevention strategies.

### 2.1 Pharmaceutic Intervention of Alzheimer's Disease

Over the past 30 years, the FDA has approved the use of three cholinesterase inhibitors (donepezil, rivastigmine, galantamine) and one NMDA receptor antagonist (memantine) for the treatment of AD. While they can enhance cognitive and memory functions, it is regrettable that they cannot halt the progression of the disease and may lead to significant adverse effects [19]. Over the years, in order to develop more effective drugs, researchers have conducted various experiments and shifted their research and development towards disease‐modifying therapies [20]. As of 2024, a total of 164 clinical trials at different stages are in progress, involving 127 drugs, most of which are centered on Aβ and Tau proteins [21].

Aβ‐directed therapeutics aim to reduce the level of Aβ in the brain parenchyma of AD patients by reducing Aβ production and increasing Aβ clearance, thereby slowing the progression of AD [22]. Approximately 30 years ago, the AN1792 vaccine became the first active immunotherapy for AD treatment but was soon terminated due to the occurrence of acute meningitis in patients during clinical trials [23, 24]. Despite many setbacks, it is gratifying that two Aβ‐targeted monoclonal antibodies (Lecanemab and Donanemab) have achieved much progress recently [25, 26]. Nevertheless, it is crucial not to disregard the most impactful adverse effect, amyloid‐related imaging abnormalities, which appear as regions of effusions or as hemorrhagic lesions, remaining a concern for the safe application of anti‐Aβ immunotherapy in aging patients [27, 28].

Because of the close correlation between Tau and the severity and symptom types of dementia, Tau‐targeted therapy has become a promising approach in combating AD. The current research direction for Tau‐targeted therapies also tends to immunotherapeutic strategies, including vaccines and antibody therapies [19, 29]. Considering the limitations of previous treatments, such as low bioavailability and the presence of the blood–brain barrier, a team led by Kayed developed a nasal spray for toxic Tau conformation‐specific monoclonal antibody‐2 (TTCM2) [30]. Through safe and non‐invasive nasal administration, it aims to effectively eliminate intracellular and synaptic Tau aggregates, thereby improving cognitive function in aged tauopathy mice. This represents an undoubtedly significant breakthrough in the field of Tau immunotherapy.

Since single‐target western drugs are unlikely to yield satisfactory curative effects, more and more scientists pay attention to traditional Chinese medicine (TCM) [31]. Many theories of TCM have been passed down and are still applied in clinical practice currently. For example, “Qi‐Fu‐Yin” “Danggui‐Shaoyao‐San” “Yi‐Gan‐San” and “Liuwei‐Dihuang decoction” can nourish Blood and strengthen organ function; therefore, to ameliorate the AD's pathologies and behavior deficits by reducing neuroinflammation and promoting neurogenesis [31, 32, 33, 34, 35].

Due to the research progress in the discovery of AD biomarkers and elucidation of its underlying pathogenic mechanisms, the development of new anti‐AD drugs has made rapid progress in recent years. However, translating these drugs into clinical practice remains a huge challenge. While there is currently no specific drug capable of halting or reversing the progression of AD, an increasing body of evidence suggests that various non‐pharmacological interventions such as dietary modification, physical exercise, and lifestyle change may help delay or prevent the onset of dementia.

### 2.2 Non‐Pharmaceutic Treatment of Alzheimer's Disease

Compared to pharmaceutic treatment, patients may have higher acceptance and better adherence to non‐pharmaceutic approaches. From the perspective of cognitive psychology, meditation practice and mindfulness can regulate mental‐psychological states and induce favorable neurostructural and neurofunctional changes that promote mental health in both healthy and clinical populations [36]. Long‐term sleep disorders exacerbate the progression of cognitive impairment in AD patients, but mindfulness‐based stress reduction may possibly reverse this process by reducing anxiety and depression, thereby delaying the decline of cognition [37].

A body of research has demonstrated that elevated levels of physical activity are associated with a decreased risk of cognitive decline [38]. Multiple meta‐analyses suggest that prolonged physical exercise can provide an improvement in cognition for older adults with memory problems [39, 40], which indicates its potential in the treatment of AD. Meanwhile, in the laboratory, researchers have found that exercise plasma reduces neuroinflammation and boosts memory in the AD mouse model [41].

Another non‐invasive therapy called Terahertz technology has recently been considered as a novel AD therapeutic intervention because of its benefits in the neurobiological field such as modulating the structure of neurons [42]. A study revealed that Terahertz waves not only alleviated AD neuropathology and neuroinflammation but also enhanced cognitive behaviors and mitochondrial function in an AD animal model [43]. This inspiring result provides a basis for Terahertz therapy as a safe alternative for AD management in the future.

### 2.3 Early Prevention for Alzheimer's Disease

AD encompasses a preclinical asymptomatic phase that can persist for about two decades, resulting in a relatively low treatment‐seeking rate. It is necessary to conduct early prevention and intervention in daily life for the elderly population. At present, experts have proposed various preventive strategies for AD including physical exercise [44], a proper diet which will be the focus of discussion next [45] and good sleep quality [46]. In addition to developing healthy lifestyles, cognitive training is thought to be an approach which exerts a positive effect on global cognition in patients with mild to moderate dementia [47]. More importantly, much evidence suggests that taking the initiative to reduce risk factors at the early stage of life can help to retain cognition better [48].

## 3 Dietary Restriction and Its Benefits on Alzheimer's Disease

**FIGURE 1:**
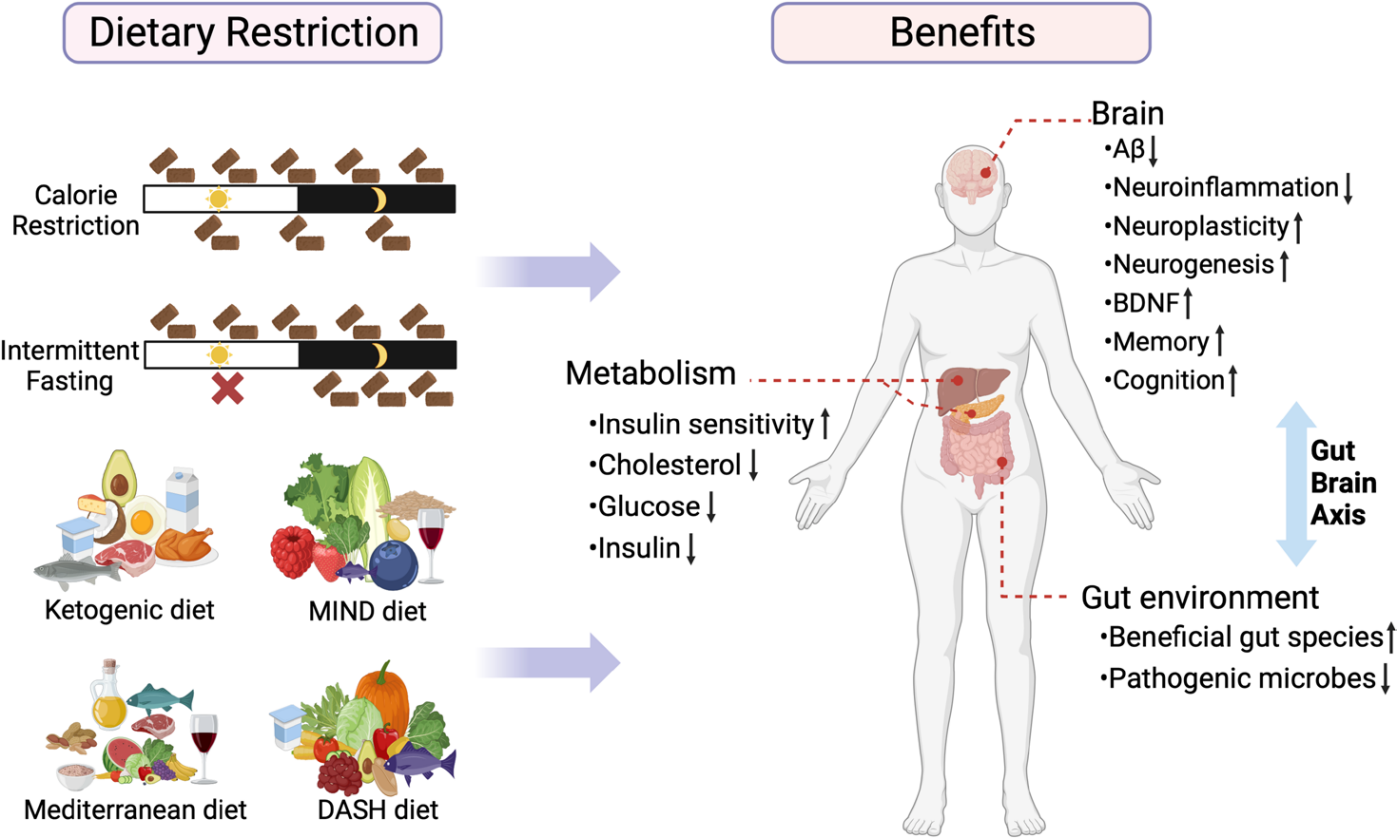
Types of dietary restriction (DR) and the beneficial effects on AD. DR can mainly be divided into several types: Calorie restriction, intermittent fasting, and four nutritional diets including ketogenic diet, MIND diet, Mediterranean diet, and DASH diet. The benefits of DR extend throughout the body, such as promoting brain health, improving metabolic circulation, and regulating the intestinal environment. In addition, the change in the gut microbiota induced by DR has a potential effect in treating AD, possibly through the gut‐brain axis. Abbreviations: MIND, Mediterranean‐DASH Intervention for Neurodegenerative Delay; DASH, Dietary Approaches to Stop Hypertension; Aβ, amyloid β‐protein; BDNF, brain‐derived neurotrophic factor. (This graphic was created in https://BioRender.com.)

Diet and nutrients play a significant role in both the prevention and alleviation of neurological symptoms. In general, DR encompasses a range of dietary interventions, including calorie and macronutrient restriction and intermittent periods of repeated cycles of short‐term fasting. Over the past few decades, DR has gained much attention in the field of aging, with substantial epidemiologic evidence and experimental data demonstrating that it may offer an attractive non‐pharmacological treatment for AD [8]. In the following sections of this review, we will discuss diverse DR protocols, including CR, IF, KD, and other dietary patterns (Figure 1).

### 3.1 Calorie Restriction

Classical CR refers to a moderate reduction in caloric intake, typically 20%–30% below the average, without inducing malnutrition throughout the entire period of dietary intervention. However, a reduction exceeding 40% may lead to negative effects [9]. CR is usually implemented in two forms: traditional CR and calorie dilution (CD). Traditional CR involves reducing a specific amount of food by a certain percentage, inevitably leading to a reduction in the intake of macronutrients such as protein, fat, and carbohydrates. This raises a new question: whether the beneficial effects of CR are attributed to the reduction in calories or the reduction in the supply of nutrients [49]. Previous studies have revealed that a low‐protein diet can significantly extend the lifespan of organisms, and this positive outcome occurs independently of calorie reduction, highlighting the unique role of protein in the context of DR [50]. Nevertheless, a recent study has suggested that energy imbalance, rather than protein intake, mediates the benefit of CR [51]. Regarding this controversy, future CR experimental protocols must meticulously consider the nutritional components and proportions of the feed utilized.

CD involves the addition of non‐digestible and non‐absorbable substances, such as fiber, to food in order to dilute its energy density, representing an alternative way of calorie restriction [49]. During the CD, mice have free access to food, whereas classical CR mice experience heightened hunger and exhibit food anticipatory activity, which is regulated by AgRP and POMC neurons [52, 53, 54]. Additionally, CR mice undergo a fasting period inevitably due to the lack of food. In a recent study, the results demonstrated that compared to CD, classical CR can more effectively extend lifespan, which indicates that hunger signals and CR‐imposed fasting time are necessary to improve health and longevity [55].

As a simple dietary regimen with relatively few side effects, CR can effectively improve cognitive function and promote overall health [56]. It is considered to be a promising prevention and treatment method for AD and even other age‐related diseases. Since McCay et al. initially conducted a CR experiment on rats in 1935, which confirmed the benefits of CR in improving cognition and delaying aging, a substantial body of research has been conducted over subsequent decades, encompassing a spectrum of organisms from unicellular to primates, and then to the largest “CALERIE” clinical trial [57, 58, 59]. Furthermore, CR has been demonstrated to rescue associative memory impairment [60], alleviate hippocampal Aβ burden [61], improve neurogenesis and neuroplasticity [62], and intervene in the autophagy pathway [63], all of which are closely related to the pathogenesis of AD. However, research conducted on 9‐month‐old PS19 mice found that CR failed to reduce p‐Tau levels. This outcome is in line with prior findings in Tg4510 models [60, 64].

Notably, the average age of AD patients is typically above 65 years, and most elderly individuals have already suffered from cardiovascular and endocrine‐metabolic‐related diseases. Casual CR intervention may lead to malnutrition and decreased immune function, potentially exacerbating health problems in turn. Therefore, when conducting clinical trials of CR, the patient's baseline health status must be taken into account to ensure the safety and feasibility of the study.

### 3.2 Intermittent Fasting

Throughout history, our ancestors were often forced into energy restrictions or fasting due to irresistible factors such as wars and disasters, which hindered the development of a daily routine of three meals plus snacks. From an evolutionary perspective, the brain operates most effectively when an individual experiences hunger and actively pursues food [65]. At present, scientists are exploring a new dietary strategy—fasting—and attempting to elucidate its health benefits, especially in the prevention and treatment of AD. To date, although there is no clear definition of IF, it is widely accepted that IF can be divided into alternate‐day fasting (ADF), the 5:2 diet or periodic fasting (PF), time‐restricted feeding (TRF) and fasting mimicking diet (FMD) according to the duration of fasting time [66]. Compared with CR, IF does not strictly limit the daily calorie intake but pays more attention to the timing of food consumption, with fasting periods ranging from 12 to 48 h. It is important to acknowledge that for most people, especially those with AD, it is often difficult to maintain a 30%–40% CR for a long time due to their physical condition, whereas IF presents a relatively safe and feasible dietary alternative.

During fasting, a metabolic shift is one of the most prominent features. In daily activities, the brain requires a large amount of glucose as its primary source of energy. However, under certain conditions, such as when the fasting duration extends to 12–36 h, almost all the glucose in the body is depleted. Adipose cells then release fatty acids into the circulation, which are converted to ketone bodies in the liver. Ketone bodies can serve as an alternative energy source to maintain brain function. This transition process from glucose to ketone bodies is known as the “G‐to‐K switch” [67, 68, 69]. In the fasted state, the brain needs to promptly adapt to this metabolic shift and maintain normal physiological functions, which can enhance cognitive function and promote brain health. Overall, IF triggers highly coordinated cellular and systemic responses, thereby enabling organisms to achieve an optimal metabolic state.

However, whether calorie intake or fasting duration plays a more essential role in brain health remains an open question. In initial research, reducing calories was often linked to fat loss [70]. Accordingly, experts mainly attributed the positive effects on neurodegenerative and metabolic diseases to weight reduction. As IF has gradually gained public attention, “starving time” and “hunger” have emerged as another core focus. An intriguing experimental observation shows that the food intake of mice subjected to a 5:2 fasting diet is almost identical to that of the control group [71]. Researchers clarify that following fasting, mice become hungry, so once they are given access to eat, their food intake increases significantly, compensating for the calorie deficit. Consequently, scientists have determined that the health benefits induced by fasting are independent of total calorie intake but are related to the duration, frequency, and timing of the fasting period. Studies have indicated that fasting can significantly improve motor and cognitive functions and decrease pathological markers in AD mice, thus demonstrating its considerable potential for therapeutic application in the treatment of AD [72, 73]. In addition, circadian disruptions and sleep disorders, which often manifest in the early stage of AD, can be alleviated through TRF. This may be attributed to the ability of TRF to modulate hippocampal gene expression and strengthen the circadian rhythms [74].

While IF offers a more flexible and suitable dietary approach compared to CR, individuals may initially experience hunger, irritability, and decreased concentration during the early stage of fasting. To mitigate these physiological discomforts, participants can adopt a gradual approach by incrementally increasing the duration and frequency of fasting over the initial few months. Despite the potential IF has shown in animal models across various disease areas, including obesity, cancer, diabetes, cardiovascular disease, and neurological disease, clinical studies mainly focus on overweight young and middle‐aged individuals. Consequently, the benefits of IF to AD patients cannot be conclusively extrapolated at this time. Further investigation is warranted to fully explore the advantages of IF and to mitigate any risks for the elderly.

### 3.3 Ketogenic Diet

Another diet strategy, ketogenic diet (KD), referring to a very low‐carbohydrate, adequate‐protein, and high‐fat diet, is widely acknowledged as an effective obesity management strategy and treatment for children with drug‐resistant epilepsy [75, 76, 77]. Apart from this, the existing proofs support the use of KD as an alternative therapy in neurodegenerative diseases, including AD, Parkinson's disease and Amyotrophic lateral sclerosis [78]. The metabolic changes induced by KD are similar to those of IF, as mentioned earlier. The very low‐carbohydrate content of KD makes the body mistakenly believe that it's in starvation mode, resulting in the conversion of fat into ketones by the liver. The ketone body (i.e., β‐hydroxybutyrate, BHB) appears to be the key factor that brings benefits in both protocols [79]. Interestingly, KD is also related to CR to some extent due to appetite suppression and a reduction in food variety. As for AD, progressive cognitive degeneration is linked to reduced uptake and dysmetabolism of glucose, while ketone bodies can replace glucose as the main energy supplier and produce more ATP for the brain [80]. Furthermore, KD has an effect on regulation at the mitochondrial level, including reducing reactive oxygen species (ROS) production, enhancing ATP concentrations, and activating mitochondrial uncoupling protein [78, 81]. Given the mitochondrial dysfunction in AD, these mechanisms may confer antioxidant and neuroprotective benefits. Both animal and human studies have shown that KD can reduce Aβ deposition [82], improve patients' daily function and quality of life [83], indicating its potential as a therapeutic intervention for AD.

Implementing KD actually requires very strict control over the proportion of macronutrient intake in order to force the body to switch metabolic states. Furthermore, for individuals who are accustomed to an Eastern diet, it is challenging to adapt to excessive “fatty meat” a day. Based on the discussion above, KD shares similarities with IF and CR in several aspects; for example, they utilize the ketone body as the primary energy source for the brain, despite it being in different ways. Therefore, unraveling the exact mechanisms and pathways through which these dietary manipulations exert neuroprotective effects is of paramount importance to recommend them for appropriate populations.

### 3.4 Other Recommended Dietary Approaches

In addition to the aforementioned three dietary patterns, several other well‐recognized nutritional diets also offer benefits for brain health, including the Mediterranean diet (MeDi), Dietary Approaches to Stop Hypertension (DASH) and Mediterranean‐DASH Intervention for Neurodegenerative Delay (MIND). Collectively, these three dietary approaches provide an extensive range of nutrients, such as Omega‐3 fatty acids, antioxidants, and vitamins, which contribute to the improvement of brain health. Observational evidence suggests that these recommended diets may reduce AD risk and prevent cognitive decline [84, 85, 86]. Nonetheless, these dietary approaches exhibit limited effects on p‐Tau levels across sexes [87], which is consistent with previous animal‐based experiments. Therefore, to elucidate the effects of DR on AD tau pathology and its underlying mechanisms, comprehensive and systematic investigations remain warranted in future research.

## 4 Exploring Mechanisms Underlying Dietary Restriction Therapy for AD

**FIGURE 2:**
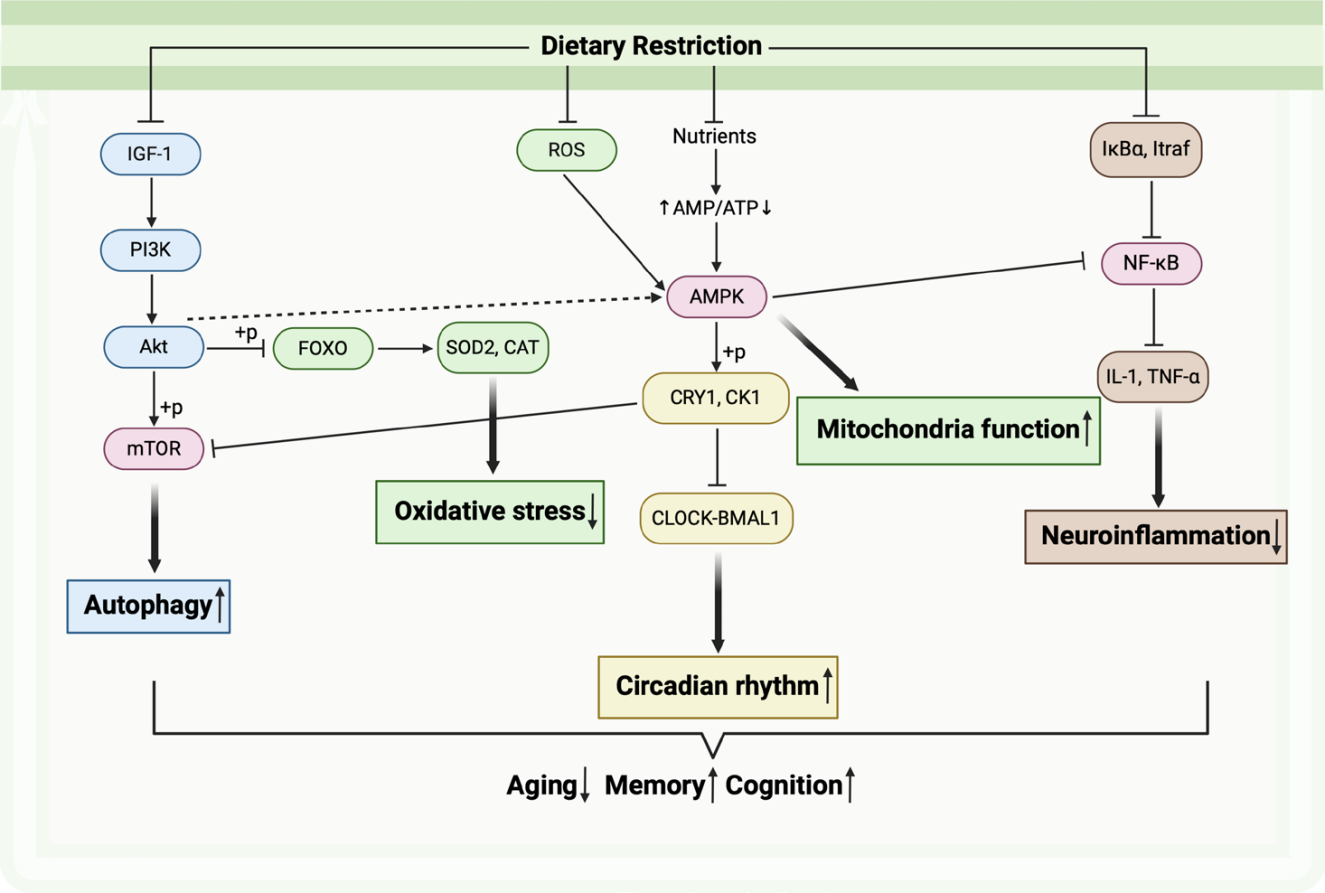
Possible mechanisms linking DR and AD. Dietary restriction exerts its beneficial effects on aging, memory, and cognition through these metabolic pathways. Autophagy, circadian rhythm, and mitochondrial function can be enhanced; oxidative stress and neuroinflammation can be alleviated. The pathways were created with the help of KEGG (https://www.kegg.jp/). Abbreviations: IGF‐1, insulin‐like growth factor 1; PI3K, phosphatidylinositol 3‐kinase; Akt, protein kinase B, PKB; mTOR, mammalian target of rapamycin; FOXO, forkhead box protein O; SOD2, superoxide dismutase 2; CAT, catalase; ROS, reactive oxygen species; AMPK, AMP‐activated protein kinase; CRY1, cryptochrome 1; CK1, casein kinase 1; IL‐1, interleukin‐1; TNF‐α, tumor necrosis factor‐α. +p, phosphorylation (This graphic was created in https://BioRender.com.)

Although many studies have demonstrated that DR has many benefits on AD, the exact mechanisms and pathways through which these dietary manipulations exert neuroprotective effects are not yet well elucidated. Researchers have proposed several hypotheses to elucidate the role of metabolism, neuroinflammation, oxidative stress, autophagy, gut microbiota, and circadian rhythm in these processes (Figure 2). Besides, Table 1 summarizes DR and AD‐related animal studies conducted within the past decade.

**TABLE 1:** Summary of DR experiments in AD.

Type	Study	Model	Intervention	Result
CR	Schafer et al. 2015 [61]	Tg2576 mice	CR for 2.8,12.5 months	Aβ↓; Psenen↓; Ps1↓
	Cox et al. 2019 [88]	Tg2576 mice	CR for 12 months	Age‐related microbiome↓
	Cogut et al. 2024 [64]	MAPT^P301S^PS19 mice	CR for 6 weeks	Spatial learning↑; cell proliferation↓; no effect on Tau and neuroinflammation
	Chen et al. 2024 [89]	APP/PS1 mice	CR for 6 weeks	Cognition↑; Aβ↓; IL‐1β↓; TNF‐α↓; ROS↓
ADF	Zhang et al. 2017 [72]	APP/PS1 mice	ADF for 5 months	Aβ↓; cognition↑
	Liu et al. 2019 [90]	App^NL‐G‐F^ mice	ADF for 1,4,12 months	Cognition↑; synaptic plasticity↑
	Lazic et al. 2020 [91]	5xFAD AD mice	ADF for 4 months	TNF‐α↑; MAPK↑; EAAT2↑; no effect on Aβ and BBB
	Li et al. 2020 [92]	3xTg‐AD mice	ADF for 3 months	Cognition↑; neuronal differentiation↑; BDNF↑
	Ye et al. 2024 [93]	3xTg‐AD mice	ADF for 5 months	Aβ↓; Tau↓; cognition↑; synaptic plasticity↑; neuronal loss↓; mitochondrial function↑
TRF	Whittaker et al. 2023 [74]	APP23 TG mice APP‐KI mice	TRF for 3 months	Aβ↓; sleep↑; memory↑; Bmi1↑
KD	Kashiwaya et al. 2013 [94]	3xTg‐AD mice	KD for 8 months	β‐hydroxybutyrate↑; cognition↑; Aβ↓; Tau↓
	Pawlosky et al. 2017 [95]	3xTg‐AD mice	KD for 8 months	Glycolytic and TCA cycle↑; mitochondrial redox potential↓; oxidized lipids and proteins↓

Abbreviations: ADF, alternate‐day fasting; Aβ, amyloid β‐protein; BBB, blood–brain barrier; BDNF, brain‐derived neurotrophic factor; CR, calorie restriction; EAAT2, excitatory amino acid transporter 2; IL‐1, interleukin‐1; KD, ketogenic diet; MAPK, mitogen‐activated protein kinase; ROS, reactive oxygen species; TNF‐α, tumor necrosis factor‐α; TRF, time‐restricted feeding.

### 4.1 Metabolism Disorder

Brain energy metabolism disorders and mitochondrial dysfunction are closely associated with the development of AD. The Lancet clearly indicates that type 2 diabetes, marked by insulin resistance, is an important risk factor for AD while glucose metabolism abnormalities are frequent among AD patients [96, 97]. Some researchers proposed that AD could be classified as a degenerative metabolic disease, specifically one mediated by insulin resistance [98]. Although insulin resistance is not the primary cause of AD, it can lead to abnormal glucose metabolism in the brain, thereby impairing ATP production and exacerbating the progression of the disease. The “G‐to‐K” switch, as observed in both IF and KD, provides BHB as an alternative substrate to glucose, which helps maintain mitochondrial function and contributes to improved cognitive performance [99].

Insulin‐degrading enzyme (IDE) is a zinc metallo‐endopeptidase that is widely distributed across various tissues in the human body and plays a pivotal role in the degradation of both Aβ protein and insulin [100]. Persistent hyperinsulinemia can lead to a significant consumption of IDE in the brain, resulting in reduced IDE levels, which in turn contributes to the abnormal deposition of Aβ and subsequent neuronal damage [101]. It is well established that CR elicits a series of complex energy metabolic changes in the body, including a 15% reduction in blood glucose and cholesterol levels, and a remarkable 50% decrease in insulin levels, accompanied by an enhancement in insulin sensitivity [102]. Consequently, CR may indirectly increase the clearance rate of Aβ by lowering insulin levels and elevating IDE content in the brain, which may potentially offer benefits for the treatment and prevention of AD.

It is worth noting that the brain metabolome is region‐specific. Furthermore, the metabolic disparities between regions are not random or independent; rather, they are closely linked to the types of metabolites, metabolic pathways, and anatomical structures [103]. While a comprehensive metabolome atlas of the mouse brain in response to fasting has recently been established [104], future research should concentrate on identifying the valuable metabolic alterations that occur in the AD brain. This is essential for gaining a deeper understanding of how DR impacts metabolism in the context of AD.

### 4.2 Neuroinflammation

Neuroinflammation, a central pathological feature of AD, originates from the chronic and sustained activation of microglia. This activation triggers an inflammatory response, leading to the production of inflammatory factors such as interleukin‐1 (IL‐1), tumor necrosis factor‐alpha (TNF‐α) and IL‐6, along with the generation of toxic products. These inflammatory factors contribute to exacerbating neuronal damage and the deposition of Aβ [105]. While the neuroprotective mechanisms of DR have not been fully elucidated, current research indicates that DR can directly modulate neuroinflammation by reducing glial cell activation and regulating the expression of inflammatory factors [106]. Furthermore, DR can indirectly mitigate the inflammatory response by reducing oxidative stress [107]. Additionally, the nuclear factor kappa‐B (NF‐κB) pathway plays a crucial role in gene expression, involving the production of inflammatory factors and the regulation of inflammatory responses [108]. DR has been shown to upregulate the I‐κBα‐chain and I‐traf proteins, thereby inhibiting the NF‐κB pathway in response to neuroinflammation [109]. Moreover, it has been reported that DR can reduce IL‐1 and TNF‐α levels in the hippocampus and increase brain‐derived neurotrophic factor (BDNF) expression, thereby providing a certain degree of neuroprotection [110]. However, a study by Lazic et al. found that ADF exacerbates inflammation and neuron deficits in 5xFAD mice, highlighting the need for more comprehensive research to fully understand its role in modulating neuroinflammation and neuroprotection in the context of AD [91].

### 4.3 Autophagy Dysfunction

Impairment of autophagy‐lysosomal function has been linked to the pathogenesis of AD, characterized by the accumulation of misfolded protein aggregates [111]. Rapamycin, a mammalian target of rapamycin (mTOR) inhibitor known for its role in modulating autophagy, has been shown to slow down aging processes and may exhibit efficacy against AD [112]. However, the side effects of Rapamycin, such as immunosuppression, hyperlipidemia, and hyperglycemia, raise concern regarding its use for promoting human longevity [113]. During periods of CR and IF, the body experiences a reduction in metabolic rate. To maintain energy balance, the secretion of insulin‐like growth factor 1 (IGF‐1) decreases, correspondingly inhibiting the activity of mTORC1 [10]. Moreover, when calorie intake is reduced, the AMP/ATP ratio increases, activating the energy sensor AMP‐activated protein kinase (AMPK). This activation subsequently leads to the downregulation of mTORC1, thereby promoting autophagy and accelerating the clearance of damaged organelles and abnormally deposited proteins [114, 115]. To date, a body of research has confirmed that DR can promote longevity through the modulation of autophagy, but there remains a lack of substantial evidence concerning its impact on AD, indicating a need for further detailed investigation [116].

### 4.4 Oxidative Stress

Given the high metabolic activity of the brain, it is particularly susceptible to oxidative stress, especially in the context of AD where there is an observed increase in oxidative stress markers accompanied by a decrease in antioxidant enzyme activity [117]. There is a close relationship between mitochondria and oxidative stress because mitochondria are the primary source of ROS. However, excessive ROS can induce oxidative stress, leading to abnormal alterations of mitochondrial dynamics [118]. Studies have demonstrated that DR can effectively combat oxidative stress in the brain by reducing the production of ROS, increasing the activity of antioxidants (such as glutathione, GSH) and antioxidant enzymes (such as glutathione S‐transferase, GST), thereby ultimately mitigating oxidative damage to biomacromolecules [119]. Moreover, fasting leads to a decrease in circulating levels of IGF‐1, which subsequently negatively regulates forkhead box protein O (FOXO)‐dependent gene transcription through the PI3K/AKT pathway [120]. This can regulate the expression of a series of antioxidant genes, such as superoxide dismutase (SOD2) and catalase (CAT) to enhance the cellular antioxidant capacity.

### 4.5 Gut Microbiota

In recent years, the “gut‐brain axis” has emerged as a pivotal concept in understanding the role of the gut microbiota in AD. Studies have demonstrated that individuals with AD exhibit alterations in the diversity of the gut microbiome [121]. Current evidence highlights a close relationship between DR, gut microbiota, and the pathogenesis of AD. An experiment conducted by Cox et al. suggested that long‐term CR may alter the gut environment in Tg2576 female mice, potentially preventing the expansion of microbes that contribute to age‐related cognitive decline [88]. Furthermore, another study demonstrated that IF could ameliorate AD pathological manifestations and improve cognitive function in 5XFAD mice [122]. This improvement is attributed to the ability of IF to modify the composition of the gut microbiome, particularly by enriching *Lactobacillus* species, which are beneficial to human health, and influencing their metabolic activity [123]. Latest research on a modified Mediterranean‐ketogenic diet revealed that this dietary pattern can improve AD‐related neurological function by shifting the gut microbiome community and microbial metabolites [124]. In summary, the change in the gut microbiota induced by DR has a potential effect in treating AD, at least in part, through the gut‐brain axis.

### 4.6 Circadian Rhythm Dysregulation

Disruptions in the sleep–wake cycle and circadian rhythm are often early manifestations of AD and are closely associated with Aβ levels in the brain [125]. Therefore, regulating the circadian clock represents a potential therapeutic strategy for AD. TRF, which aligns more closely with the circadian rhythm compared to ADF or PF, has demonstrated greater benefits in improving sleep quality and restoring activity rhythm in AD mouse models [74]. Nevertheless, direct research on TRF related to AD is limited, and the specific mechanism remains unclear. To some extent, AMPK can be considered a nutrient sensor that is activated under conditions of nutrient deficiency, such as during TRF. AMPK mediates the inhibition of the mTOR pathway by phosphorylating cryptochrome 1 (CRY1) and casein kinase 1 (CK1), both of which play roles in enhancing the stability of circadian rhythm [126]. Concurrently, the activation of AMPK can modulate the expression of genes associated with circadian rhythm, such as DBP, PER3, and NFIL3 [74, 127].

## 5 Dietary Restriction and Brain Health

It is well accepted that DR can mitigate symptoms of neurological disorders, exerting a positive neuroprotective effect by upregulating specific gene expressions [128]. A recent study has identified a key component in the cellular mechanism by which DR extends lifespan and protects the brain—the mtd/OXR1 gene [129]. Under DR conditions, mtd/OXR1 can sustain reverse transcriptase activity which contributes to the preservation of neural health and the delay of the brain aging process.

Adult hippocampal neurogenesis (AHN) is the process by which damaged neurons are replaced and neuroplasticity is enhanced [130]. AHN plays a critical role in preserving cognitive function and promoting brain health. With the increase of age, the decline of AHN is associated with certain neurodegenerative diseases and brain dysfunction. Previous work has generally reached a consensus that IF, FMD, and CR promote AHN and cognition [131, 132, 133], while the 5:2 diet does not increase AHN nor enhance memory performance [134]. The disparities observed in these outcomes indicate that scientists should fully understand the benefits and mechanisms of different DR paradigms.

Findings from observational and clinical trials suggest that DR may offer protective benefits against cognitive decline and promote brain health in the older population. We summarize some DR human studies relating to brain disorders in the last 5 years (Table 2). These results will be extremely helpful to elucidate the importance of specific dietary approaches and their potential side effects in order to recommend appropriate strategies for individuals. However, existing findings predominantly focus on healthy aging populations. It is noteworthy that the pathological processes underlying AD are distinct from normal aging trajectories. While DR exhibits robust neuroprotective efficacy in AD animal models, direct extrapolation of these outcomes to AD patients requires rigorous validation.

**TABLE 2:** Summary of DR human studies.

Type	Study	Population	Intervention	Result
MIND	Barnes et al. 2023 [135]	Older adults	MIND+mild CR for 3 year	No changes in cognition and brain MRI
	Huang et al. 2023 [136]	Middle‐aged and older adults	MIND for 3 year	Cognitive function↑; cognitive decline↓
	Li et al. 2024 [137]	Older adults	MIND until death (a longitudinal cohort)	Cognitive decline↓; odds of dementia↓
MeDi	Hoscheidt et al. 2022 [138]	Adults (age range: 45 to 65) with NC or MCI	Med‐diet or West‐diet for 4 weeks	Powerful effects on AD pathology and cerebrovascular indices
	Kaplan et al. 2022 [139]	Abdominally obese/dyslipidemic participants	HDG, MED or Green‐MED diet for 18 months	Insulin sensitivity↑; brain atrophy↓
	McLeod et al. 2023 [140]	Older, African American obese adults	Med‐Diet or IWL for 8 months	No changes in gut microbiome
CR	Leclerc et al. 2020 [141]	Healthy non‐obese adults	25% CR for 2 years	Working memory↑
IF	Ooi et al. 2020 [142]	Adults (age > 60) with MCI	IF for 36 months	BMI↓; blood pressure↓; cognitive function↑; MDA↓; DNA damage↓; insulin↓
	Zhao et al. 2025 [143]	AD patients	TRF for 4 months	Cognitive function↑; executive function↑
KD	Phillips et al. 2021 [83]	AD patients	KD for 12 weeks	Daily function↑; life quality↑
	Fortier et al. 2021 [144]	Adults (age > 55) with MCI	KD for 6 months	Executive function↑; memory↑; language↑

Abbreviations: CR, calorie restriction; Green‐MED, MED higher in polyphenols and lower in red/processed meat; HDG, healthy dietary guidelines; IF, intermittent fasting; IWL, intentional weight loss; KD, ketogenic diet; MCI, mild cognitive impairment; MDA, malondialdehyde; MeDi, Mediterranean diet; MIND, Mediterranean‐DASH Intervention for Neurodegenerative Delay.

## 6 Conclusion and Prospects

The effect of DR on brain health and neurodegenerative diseases is complex and still not fully understood. Research conducted over the past few decades suggests that DR could be an effective non‐pharmacological treatment for AD. It holds promise as a behavioral paradigm for mitigating cognitive decline, alleviating AD pathology, and improving overall health, thereby potentially delaying the onset and slowing the progression of AD. As we have discussed in this review, several mechanistic hypotheses have been proposed. By modulating metabolic pathways, DR can reduce inflammatory responses, counteract oxidative stress‐induced damage, and enhance the autophagy process. Furthermore, DR has been shown to reshape the gut‐microbiota‐metabolites‐brain axis, which facilitates its neuroprotective effects against AD. Given the prevalent circadian disruptions observed in AD patients, DR emerges as a potent modulator of circadian rhythms, capable of normalizing brain transcription and ameliorating disease progression. While the above mechanisms individually exhibit distinct therapeutic potential, the hierarchical or synergistic relationships between these pathways remain incompletely understood. To elucidate these intricate interactions, future investigations employing integrative multi‐omics methodologies (metabolomics, lipidomics, proteomics, etc.) will prove indispensable for unraveling the intricate network of molecular crosstalk.

Nonetheless, the transition of DR from laboratory research to clinical implementation remains a challenge. A major impediment is that current research models such as yeast, drosophila, and mice exhibit substantial differences from humans in various aspects, including metabolism, lifespan regulation, and disease progression, making it difficult to directly apply experimental protocols to humans. Specifically, the absence of the Preiss‐Handler pathway in yeast renders this model inadequate for studying human‐specific metabolic adaptations to nutritional interventions [145]. Also, differences between transgenic mice and humans represent a pivotal barrier to the clinical translation of DR. While transgenic mouse models can recapitulate key AD pathology, gene overexpression might trigger non‐specific phenotypes irrelevant to AD progression, which are different from human clinical manifestations [146]. Additionally, even when implementing the same DR approach, slight differences in experimental details, such as the time of food delivery (morning vs. evening), the nutritional composition of the food, and the initiation and duration of the experimental period, can exert notable influences on the outcomes. In humans, factors such as gender, age, genetics, and lifestyle habits pose significant challenges in exploring the effects of DR. Given these limitations, future research in this field should focus on gaining a deeper understanding of the molecular mechanisms of DR, with the aim of developing safe and effective Calorie Restriction Mimetics (CRMs). These CRMs would circumvent the necessity for individuals to reduce daily calorie intake or nutrients. Instead, CRMs might emerge as a novel class of pharmaceuticals, providing novel therapeutic avenues for the management of neurodegenerative disorders.

In conclusion, DR exhibits promising preventive and therapeutic effects on the pathological manifestations of AD. However, for individuals with AD, maintaining a prolonged low‐calorie diet may induce the risk of malnutrition, potentially exacerbating cognitive decline and physical debility. Consequently, there is an urgent need to conduct rigorous and extensive scientific research, coupled with longitudinal clinical assessments, to ascertain the efficacy and safety of DR in AD patients. In parallel, due to the complexity of AD, patients at different disease stages and with varying physical conditions may exhibit diverse responses to DR. This suggests that DR may not represent a universally applicable therapeutic modality. Therefore, the implementation of personalized intervention protocols is essential when administering DR regimens to AD patients. Such endeavors will facilitate a comprehensive understanding of its therapeutic benefits and potential adverse consequences, thereby ensuring the rationality and safety of the treatment regimen.

## Conflicts of Interest

The authors declare no conflicts of interest.

## Data Availability

Data sharing not applicable to this article as no datasets were generated or analysed during the current study.
